# The Behavior of Consumers and Producers of Food of Animal Origin and Their Impacts in One Health

**DOI:** 10.3389/fvets.2021.641634

**Published:** 2021-06-14

**Authors:** Natália Maramarque Nespolo

**Affiliations:** Veterinary Medicine Nucleus, Federal University of Sergipe, Nossa Senhora da Glória, Brazil

**Keywords:** food, zoonosis, health, education, animal products

## Abstract

Most people consume animal foods, for example meats, but few are concerned with the quality and origin of these products. Many studies point out hygiene problems of these foods after production; however, the lack of knowledge of the consumers of animal products about the importance of hygienic-sanitary control during the production process can lead them to a bad choice when buying these products and, consequently, expose themselves to the risk of acquiring many diseases, such as zoonosis. In this perspective, the objective of this work is to reflect about the consumers' role in the production of safe food of animal origin and to show that the population's health education is necessary and urgent. Only by helping the consumers to obtain knowledge about the production of animal products origin will there be a change in consumption habits, preventing the ingestion of contaminated foods that can cause damage to human health and to the environment, consequently, promoting one's health.

## Introduction

Protein from animal sources, especially meat, is an important source of nutrition for many people around the world, and to produce them in a sustainable way is one of the challenges in the coming decades (1). Foodborne diseases are a serious global health problem (2), and in developing countries, most people are not concerned about the norms in the processing and packaging of the food (3) that they will consume and/or offer to other people to eat (4, 5). Due to this carelessness or lack of knowledge about safety production and handling of food of animal origin at the time of purchase, people do not know whether they are buying clandestine or manufactured products that have a good hygienic-sanitary standard supported by legislation and guarantee safe consumption.

Some questions about food of animal origin are important and should be considered at the time of purchase or consumption, such as what would be the origin of the food that I am buying? Do I search and know how to identify in the label if the products of animal origin have been inspected and produced by hygienic-sanitary standards? Would I buy the product if I knew that it is harmful to health and to the environment just because it is cheaper? Do I know the diseases that I can get if I consume a contaminated product from animal sources?

Unfortunately, most people do not have the answer or the habit of thinking about these questions, mainly in developing countries. In Latin America, there are many researches related to the profile and behavior of the consumer of food from animal sources, and it showed that most of the people do not worry about the animals' sanitary conditions and the good hygienic practices during the production process (2, 4–10). They care most about the color, tenderness, and price of the food, and in some regions, it is explained by the low education of the population, including people who are analphabet (4). In Brazil, a study about consumer perception of risks associated with food, safety and traceability showed that some consumers are concerned about health risks linked to animal products, but they have a wrong idea about the concept of hygienic-sanitary factors, as could be seen in the phrases like “A product that is not so perishable, such as honey, could buy both from the farm and from the supermarket, as it does not offer such a great risk” and “the cheese on the farm tastes better than the supermarket” (6). Therefore, if the consumers do not know about the hygienic and sanitary aspects of the food of animal origin that they will eat, will the producers and farmers really do care about it?

In addition to the lack of knowledge, the consumers are also false-information hostages about the product they will purchase and/or consume (6, 10) and end up not making the best choice at the market. Disinformation is often spread by famous digital influencers (11) who are not experts in the production of food of animal origin. This lack of scientific knowledge of the consumers ends up harming their own health and the economy, because in addition to running the risk of eating contaminated food, many also stop eating meat and other animal products due to the negative influence of lay people on the subject or motivated by ethical and moral issues, like their own beliefs in animal welfare and environmental impact of production systems (10, 12, 13).

Thus, the objective of this work is to reflect about the role of the consumers and producers in the production of safe food from animal sources and to show the necessity and urgency of education on health, and to have changes in people's beliefs and consumption habits. Thus, it will help people to know more about the proper production of food of animal origin and, consequently, to choose better products at the time of purchase, thereby preventing the ingestion of contaminated foods that can cause human diseases and promoting producers to have good manufacturing practices, promoting one's health.

## Hygiene

Basic personal hygiene habits, despite being simple, still are extremely important and must be acquired in childhood because children, besides growing up with these habits, are good disseminators of information to other people (14). The new Coronavirus pandemic highlighted the importance of hygiene habits in early childhood education (15). A primordial habit to produce food safely is to wash hands properly, especially before handling (3, 16). Food handlers are often associated with foodborne disease outbreaks and are estimated to contribute 7–20% of the outbreaks (17). Improper poor personal hygiene of food handlers contributes most to disease incidence (16).

Personal hygiene habits are also different between genders, since men are less likely to wash their hands after going to the bathroom than are women (17). This is an important data for producers of food of animal origin, because most people who work in the production area are men, as shown in a research made in Brazil about employment and occupational accidents in the slaughterhouse (18). The simple act of washing hands can prevent many diseases carried by food (16, 17).

Another bottleneck is in practicing hygiene during the production of food from animal sources (19), that is, whether the producers are practicing correct sanitation as regards the environment, the equipment, and the materials to not allow food contamination (3, 17). The use of potable water and the constant monitoring of its quality in food manufacturing are extremely important (3, 17) but are largely overlooked by consumers. Imagine if the cheese you eat was made with water from a river polluted by sewage, would you eat it?

Some people in developing countries eat fecal bacteria for a long time, and people and the municipal government do anything to change it, as shown in the state of Sergipe in Brazil. In 2008, a study showed the results of the analyses of 60 rennet cheeses commercialized in Aracaju city; the samples presented *Salmonella* spp. (26.7%), coagulase-positive Staphylococcus (46.7%), total coliforms (93.3%) with values from 8.0 × 10^2^ to 1.23 × 10^4^ NMP/g, and thermotolerant coliforms from 2.72 × 10^2^ to 1.12 × 10^3^ NMP/g (20). In 2019, another research was carried out in the same city with 18 rennet cheeses in which, after 11 years, the cheeses continued to be commercialized with coliforms (3.8 × 10^3^ and 1.1 × 10^7^ CFU/g), *Staphylococcus* spp. (8.0 × 10^2^ to 1.2 × 10^6^ CFU/g), *S. aureus* (50%), pathogenic *E. coli* (16.67%), and *Salmonella* (5.55%) (21).

Other factors, such as technological ones, also help in preserving perishable products, for example the room's temperature control (3, 16, 19). Together with the poor personal hygiene of food handlers, improper holding temperature also contributed most to disease incidence (16). Commonly, the researchers are concerned about the hygienic conditions of the products during or after the sale (3, 16), which can also beget foodborne diseases due to incorrect handling (2), but it is substantial to care about the flowchart production before the sale too. Thus, it is important to use the farm-to-fork strategies to produce hygienic food and to keep the consumers and the planet healthy (17).

**Figure 1:**
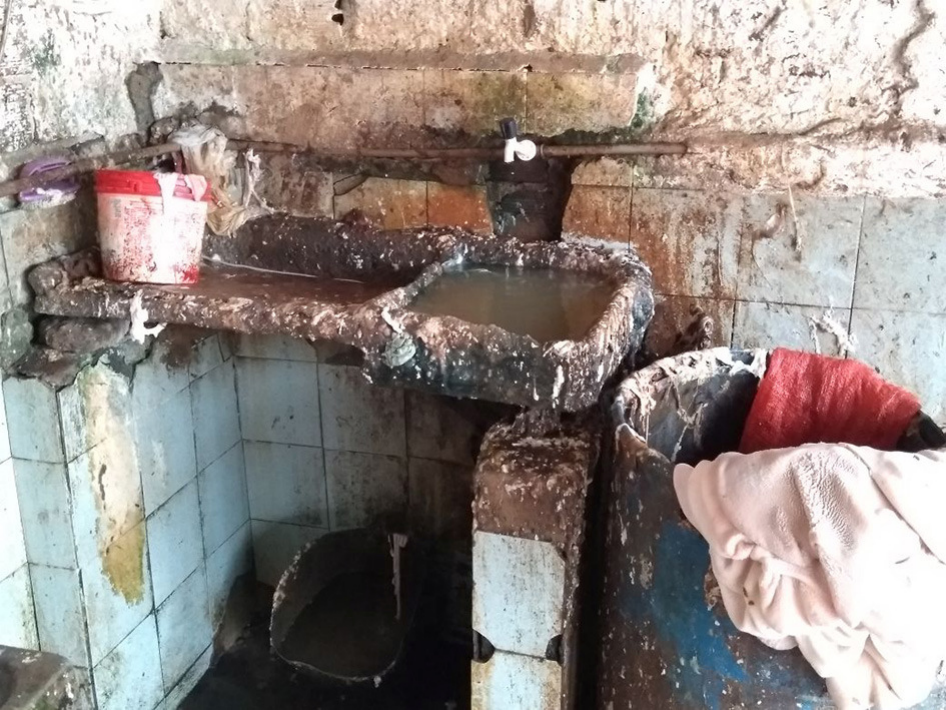
Absence of quality-control programs of a clandestine bovine slaughter, showing a disorganized, and dirty production (24).

All details, even the minute ones, must be carefully considered to produce food from animal sources safely. For this reason, there are quality-control programs in industries, such as good manufacturing practices (GMP) and hazard analysis and critical control points (HACCP) (17, 19, 22), that allow the organization and standardization of the production. Hence, it ensures equal hygienic and sanitary conditions during the manufacturing process, and it allows the identification of probable dangers to reduce them to acceptable levels or cut them out (17, 19). However, few people know about these programs, and they are not present in the clandestine production of food from animal sources. The illicit food process is totally disorganized and dirty, leaving out the conduct of some (or several) precautions and allowing the contamination of the food produced (23) (Figure 1).

## Animal Health and Welfare

To produce food safely for human consumption, another bottleneck is in selecting healthy animals. Most people know that veterinarians work at farms, but they do not know that these professionals must be present in food industries also. Inside the slaughterhouse, the veterinarians are responsible for the inspection of the animals (25), and if the animal or the carcass shows any sign of disease or contamination, they will determine if the meat could be eaten, if it needs to be processed (25), for example by heat or salt treatment (17), or if it shall be condemned totally, preserving people's and environment's health. At clandestine slaughterhouses, the animal health status is not identified (23).

In Brazil, even though the federal inspection registration of the food from animal sources was considered important by the interviewees in a city of Piauí state at the time of purchase, the carcasses do not go through any type of inspection. Due to lack of options, most interviewees buy pork meat in butchers with precarious hygienic conditions, in direct contact with microorganisms in the environment and objects, without any type of refrigeration, and at the end of the day, the meat goes to the freezer (12).

Animal welfare is an important issue that is being increasingly expanded in the world, but it still needs to be more divulged. In Mexico, people conveyed a high level of empathy with animal feelings and emotions; however, they clearly demanded more information and regulations related to farm animal welfare (4). The Mexican consumers mostly agree that animal welfare should be part of the teaching education programs in primary schools (4). Some Brazilians believe that pigs go through all kinds of discomfort during production and slaughter (12). Free-range chicken meat is preferred by Brazilian consumers (50.32%) when compared to caged chicken (36.13%), and people who buy chicken meat from the caged system do it because it has the lowest price (31.61%) (10). The preference for free-range chicken meat by 40.65% of the consumers was due to its appearance and by 23.23% because they think that it contains more nutrients than caged chicken meat (10).

If the animal is not well-treated and it does not have the five freedoms guaranteed, it is impossible to get good production and quality meat (10, 26). It could be endorsed as dark, firm, and dry (DFD) or pale, soft, and exudative (PSE) meat, which appears when animals are submitted to chronic or acute stress before slaughter, respectively (26, 27). However, many companies are still resistant to these standards, as they are associated with rising cost facilities and labor training, reflecting on the cost of the final product (10).

## Human Health Risk

Food spoilage is different from food contamination (22). Spoiled foods are those that present changes in color, odor, flavor, and/or texture because they contain deteriorating microorganisms, being rejected by consumers (17, 22). Contaminated foods usually have pathogenic microorganisms, but the characteristics (color, odor, flavor, and texture) of the food are not altered and are naturally consumed (22). People will notice after consumption and usually present symptoms such as belly pain, vomiting, and diarrhea (22). Foodborne diseases can be much more serious than a short episode of gastroenteritis, with the possibility of residual (chronic) symptoms and the risk of death, especially in elderly and immunosuppressed patients (17). Thus, if the animal has a disease or if the food is not manufactured hygienically, pathogenic microorganisms may be present in the product and the consumer may acquire a disease. Still, food can be contaminated by physical hazards, such as nails, wood, and plastic, among others, or chemical hazards, such as detergents and pesticides, among others; however, all the hazards can be controlled by applying good manufacturing practices (22). In a research made in Mexico about consumers' perceptions and attitudes toward farm animal welfare and willingness to pay for welfare friendly meat products, they related that the three main risk factors associated with conventional animal foods were residues of antibiotics, hormones, and pathogens (4).

When the production of the food from animal sources is not carried out correctly or when it is illicit, the consumers' chances to acquire a foodborne disease are high, because the products that do not have adequate quality control are dangerous and harmful to human health. Among the most frequent diseases transmitted by food of animal origin are teniasis, brucellosis, tuberculosis, listeriosis, salmonellosis, toxoplasmosis, botulism, staphylococcal intoxication, hemolytic-uremic syndrome, campylobacteriosis, and diphyllobotriasis (15–17). It is estimated that 75% of emerging human diseases are zoonosis (28), and 20% of all human illnesses and deaths are associated with endemic zoonosis (2). Epidemics and even pandemics have their etiological agents linked to the consumption of food of animal origin, and although not yet confirmed, the coronavirus pandemic may have occurred due to the consumption of bat soup (15). In many circumstances, only a small number of people seek medical help, and not all are investigated. Even when the country has infrastructure for reporting data, only a small portion of foodborne diseases are reported to the authorities (17).

These diseases harm the economy of countries due to the work absenteeism, production and tourism decrease, and high expenses with hospitalizations and health treatments (2, 3, 17, 29). Foodborne diseases are a high economic burden (2). According to the US Food and Drug Administration, foodborne illnesses have a total economic impact of 5 to 17 billion dollars (17). But why is the habit of consuming food from animal sources with good hygienic-sanitary control still not taken as seriously as it should be? How much is the consumer willing to pay or lose to obtain food safety?

## Environment Risk

To produce food whose raw material is an animal, the treatment and correct disposal of residues must be considered so that they do not harm and pollute the environment (19, 30). When a product is clandestine, the producers do not care about the environmental preservation; for example, the blood and the rest of the carcasses are exposed to the environment, causing the presence of synanthropic animals and contamination of the soil, rivers, and groundwater (23).

**Figure 2:**
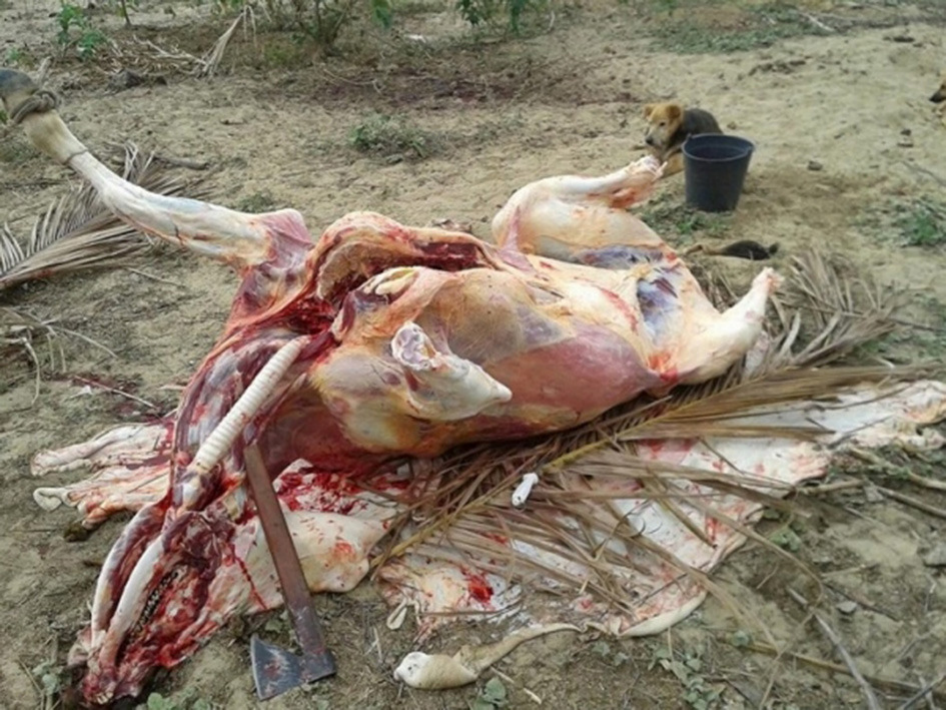
Environmental damage and bad hygienic conditions of a clandestine bovine slaughter (31).

In addition, in illicit slaughter the animal stays in an open place, under environmental temperature, with the presence of other animals such as carnivorous birds and dogs (Figure 2) that lick the carcass during the slaughter or feed on the remains of the carcass (23) and can be infected if the slaughtered animal is sick. The contact of the blood of a sick animal with open wounds or mucous membranes of the slaughterers who perform the slaughter also propitiate the spread of zoonosis, particularly the ones considered as neglected zoonosis associated with infrastructure problems and low socioeconomic status (32).

## Discussion

Safe foods are those that do not contain harmful agents or substances in quantities that may cause health problems or damage to the consumer; in other words, they are those that do not offer health hazards and guarantee the consumer's integrity (17). For this, it is necessary to have a systematic and proactive approach that minimizes food contamination from the farm to the fork (2, 5).

Correct food handling is essential to avoiding contamination by microorganisms (16, 22), and knowledge about the production of food of animal origin is essential for the consumers to choose quality and safe products for consumption (33), ensuring the preservation of their own health and the environment that they live. Many people think that it is enough to cook the food or to look for fresh food that is not dangerous and does not damage their health (16). During the cooking process, while vegetative microorganism cells are killed, the spores produced by some microorganisms, such as *Bacillus cereus* and *Clostridium perfringens*, can survive (17).

Have the consumers ever wondered which their responsibilities are when buying and/or consuming food? What their role would be in the sale of inspected animal products and in the reduction or extinction of the commercialization of clandestine products? The price of clandestine products is usually cheaper, but the products do not have the same quality as manufactured products within legal standards (33), for example, the comparison between choosing a clandestine and an inspected animal-origin product to the acquisition between a false and an original electronic product. It is a similar situation because the consumer's choice will depend on their knowledge about the factors that are linked to the production.

Moreover, it will depend on the consumer's interest in knowing how that product is being manufactured, especially about the quality of the raw material that makes it up, the production processes, the work conditions, and the damage caused to the environment. It should consider not only the assessment of risks but also technical possibilities, consumers' attitude/behaviors, and cost–benefit analysis (2). Are the production costs similar? The knowledge of the production factors and a brief analysis of the costs (19) and the work conditions, carried out in an ethical manner, would certainly help the consumers to choose the best option to purchase safe food.

The lack of consumers' awareness is a big problem (16, 17), because consumers are vulnerable to receiving any type of information, including false information (fake news) that is destructive in all areas, as it distorts what is real, it is easily and quickly spread on social media, and it gains great repercussions. For example, in Brazil, there is a myth that the color of the eggshell is related to good or bad nutrition contents and some people do not eat chicken meat because they believe that it contains hormones and antibiotics, so low consumption of both meat and eggs is associated with false information about poultry feed and the production system (6, 10).

The consumption of pork meat among Brazilians is still low and mostly linked to preconception due to lack of information about the change in Brazilian pig farming and for believing that pork has a high fat content and that it is bad for health (12). In an inquiry about the key aspects considered by consumers in the purchase and consumption of pork in Piauí/Brazil, 74% of the interviewees answered that pork has the highest level of disease transmission (12). The lack of information is also the biggest barrier to the acquisition and consumption of products in terms of wellbeing (12).

The power that digital influencers have on the consumers' lives, especially those of the Z generation, is enormous (11, 34), because they only indicate a product and the people who follow them will buy it, believing in their theories that have no scientific meaning or without making a good reflection. Some people may decrease or stop eating meat (10) and acquire anemia due to the misinformation transmitted by the influencers, as they often believe that animals feel pain when slaughtered, but do not even know what animal stunning is. For example, in Brazil, it was observed that the consumption of pork meat is popular, but it is still not highly or frequently preferred, which may be linked to the myths related to the product, and this consumption may even increase with the proper clarification of the main issues, fat content and sanitary preparations (12). Therefore, greater enlightenment is necessary for the awareness of the population, and the change requires investments in marketing, which encompasses the entire meat production chain, demystifying the negative image aggregated from its production to consumption.

The lack of consumers' knowledge affects the economy and people's lives, because when there is no awareness about the existence of potential problems with food, consumers end up eating a significant amount of contaminated food and become ill (17). In addition, clandestine slaughter favors the theft of domestic animals and the extinction of wild animals. In many developing countries, hunting and the consumption of meat from wild animals are also common cultural practices and increase the risk of zoonotic transmission (28). In Brazil, the sale and consumption of shark meat in the Amazon region will expose consumers to potentially harmful levels of inorganic arsenic (iAs) and mercury (Hg), as well as contribute to the population decline of species including those that are currently categorized as threatened (8). Therefore, it is important to know better the consumers' perception about food safety, because it can influence, along with other socioeconomic and demographic variables, the choice of food to be consumed and contribute to the effectiveness of the legislation to be implemented (6).

The low or absent surveillance in production and commercialization of food from animal sources is also a huge problem; however, there will never be enough inspectors if the consumers continue with the same buying and consuming habits. At this point, it is important to raise more questions to reflect on, such as the following: Is fake news or the lack of trade supervision of these products so important if the consumers obtained the necessary knowledge and changed their consumption behaviors? Why is there a commercialization of clandestine animal products? How much do foodborne diseases cost for the public coffers?

The economic basis is the law of supply and demand (35), so if consumers are aware of the scientific knowledge that the food of animal origin they are going to buy can endanger people's health, they would certainly not make the purchase of it, and naturally, they would report the place of sale to the competent authorities, hence facilitating their work and having no need to hire an enormous number of inspectors or increase the surveillance at production and commercialization. Even so, there would be a decrease in the supply of clandestine animal products. For it to happen, it is necessary to implant knowledge through health education of the population and, thus, to modify cultural and old behaviors, because when they are rooted, they are exceedingly difficult to be modified.

In underdeveloped countries, like Brazil, people, especially the older ones, say that the situation of consumption of food of animal origin has always been the same and that no one ever died from eating clandestine products, creating the popular saying “what does not kill, makes you fat.” Another popular thought among people is that the consumption of these products can cause only a belly pain (referring to diarrhea), due to the lack of information about foodborne diseases.

In 2025, more than one billion people in the world will be elderly and more than two-thirds of them will live in developing countries (17). Population growth means an increased risk of foodborne illness, and it is not surprising that, in some countries, one in four people is at risk of contracting a foodborne disease (17). In the Caribbean region, despite undertaking limited surveillance on foodborne diseases, records related to bacterial foodborne zoonoses in food-producing animals and their associated epidemiological significance are poorly documented, giving rise to concerns about the importance of the livestock, food animal product sectors, and consumption patterns (2). It is recognized and pointed toward the relevance of pursuing a holistic One Health approach, with interdisciplinary engagement (2).

According to the World Health Organization (WHO), One Health is “an approach to designing and implementing programs, policies, legislation and research in which multiple sectors, communicate and work together to achieve better public health outcomes” (36). According to the United Nations Food and Agriculture Organization (FAO), the World Organization for Animal Health (OIE), or the Centers for Disease Control and Prevention (CDC), health outcomes depend on food safety, control of zoonoses, and combating antibiotic resistance while recognizing the interconnection between people, animals, plants, and their shared environment (36). The One Health concept has been extended beyond public health to include the ecological and environmental dynamics of disease in system-based frameworks such as Planetary Health and Eco-Health (36).

In summary, it is not enough for only inspection to be made in the animals and carcasses at slaughterhouses as well as surveillance in food sale, if there are still uninformed people who buy illegal or manufactured products without good hygienic-sanitary conditions. In the food chain, the production of safe food is everyone's responsibility (17). The moment the consumers know more, they will also be able to demand more quality and will become supervisors as well, for the benefit of their own health.

For this, education on health of the population is urgent and necessary to change the consumers' behaviors (2, 3), especially for children, so they can grow up with this knowledge, changing the old population's cultural habits and creating a future with roots fixed in scientific knowledge. In this way, foodborne diseases, which are underreported and neglected in underdeveloped countries (2), could be prevented and would support the world's economy. Global food safety is a shared responsibility.

## Data Availability Statement

The original contributions presented in the study are included in the article/supplementary material, further inquiries can be directed to the corresponding author/s.

## Author Contributions

The author confirms being the sole contributor of this work and has approved it for publication.

## Conflict of Interest

The author declares that the research was conducted in the absence of any commercial or financial relationships that could be construed as a potential conflict of interest.
